# Investigation into the role of carboxylic acid and phenolic hydroxyl groups in the plant biostimulant activity of a humic acid purified from an oxidized sub-bituminous coal

**DOI:** 10.3389/fpls.2024.1328006

**Published:** 2024-04-29

**Authors:** Richard T. Lamar, Jason Gralian, William C. Hockaday, Maria Jerzykiewicz, Hiarhi Monda

**Affiliations:** ^1^ R&D Department, Huma, Inc., Gilbert, AZ, United States; ^2^ Department of Geosciences, Baylor University, Waco, TX, United States; ^3^ Faculty of Chemistry, University of Wroclaw, Wroclaw, Poland

**Keywords:** biostimulant, humic acid, carboxylic acids, phenolic hydroxyls, quinones, semiquinone radicals

## Abstract

**Introduction:**

Humic substances (HS) are increasingly being applied as crop plant biostimulants because they have been shown to increase plant productivity, especially under environmentally stressful conditions. There has been intense interest in elucidating the HS molecular structures responsible for eliciting the plant biostimulant response (PBR). The polar and weakly acidic carboxylic (COOH) and phenolic hydroxyl (ArOH) functional groups play major roles in the acid nature, pH dependent solubilities, conformation, and metal- and salt-binding capabilities of HS. Reports on the role played by these groups in the PBR of HS found growth parameters being both positively and negatively correlated with COOH and ArOH functionalities.

**Materials and methods:**

To investigate the role of COOH and ArOH in HS biostimulant activity we used a humic acid (HA), purified from an oxidized sub bituminous coal to prepare HAs with COOH groups methylated (AHA), ArOH groups acetylated (OHA), and with both COOH and ArOH groups methylated (FHA). The original HA was designated (NHA). The four HAs were subjected to elemental, 13C-NMR, FTIR, and EPR analyses and their antioxidant properties were assessed using the trolox equivalents antioxidant capacity assay (TEAC). ^13^C-NMR and FTIR analysis revealed significant alkylation/acetylation. To determine the effects of alkylating/acetylating these functional groups on the HA elicited PBR, the HAs were evaluated in a plant bioassay on corn (*Zea mays L.*) seedling under nutrient and non-nutrient stressed conditions. Treatments consisted of the four HAs applied to the soil surface at a concentration of 80 mg C L^−1^, in 50 ml DI H2O with the control plants receiving 50ml DI H_2_O.

**Results:**

The HA-treated plants, at both fertilization rates, were almost always significantly larger than their respective control plants. However, the differences produced under nutrient stress were always much greater than those produced under nutrient sufficiency, supporting previous reports that HA can reduce the effects of stress on plant growth. In addition, for the most part, the HAs with the alkylated/acetylated groups produced plants equal to or larger than plants treated with NHA.

**Conclusion:**

These results suggests that COOH and ArOH groups play a limited or no role in the HA elicited PBR. Alternatively, the HA pro-oxidant to antioxidant ratio may play a role in the magnitude of the biostimulant response.

## 1 Introduction

Humic substances (HS), alone or in combination with inorganic fertilizers, are becoming increasingly used as crop plant biostimulants. For commercial purposes, HS are primarily obtained from brown coals, lignites, oxidized lignites (i.e., leonardites), and oxidized sub-bituminous coals. Upon application, HS elicit a metabolic reprogramming (Roomi et al., 2018) that is caused by a modified stress response in plants, which is very similar to the responses caused by abiotic stresses (Lamar, 2019). Under the right conditions, the response is a eustress, or beneficial stress response. For example, the metabolic reprogramming includes upregulation of important metabolic processes like glycolysis and the tricarboxylic acid cycle (Conselvan et al., 2018), the antioxidant defense (Garcia et al., 2012; Roomi et al., 2018), and nutrient uptake systems (Quaggiotti et al., 2004), and also includes phenotypic changes, primarily in root system architecture, which results in the development of significantly more highly ramified root systems (Nardi et al., 2021 and references therein).

HS have been used for centuries in agricultural and horticultural applications. Their primary use has been to ameliorate the effects of abiotic stresses that occur on 90% of arable lands and cause 70% of food crop yield losses (Waqas et al., 2019). However, HS have been applied as biostimulants and soil amendments using a “black box” approach, i.e., used without knowledge of the chemical structure(s) that is responsible for eliciting the plant biostimulant response (PBR). In order to potentially optimize the design, effectiveness, and consistency of plant response to HS-based products, the chemical structure(s) that is responsible for elicitation of the PBR must be identified. Many studies designed to elucidate the chemical nature of the HS-elicited PBR have been published (Piccolo et al., 1992; Canellas et al., 2008; Aguirre et al., 2009; Canellas et al., 2009, 2010; Dobbss et al., 2010; Muscolo et al., 2010; Schiavon et al., 2010; Canellas et al., 2011, 2012; Aguiar et al., 2013; David et al., 2014; Sleighter et al., 2015; Garcia et al., 2016). These studies have included applications of different types of un-fractionated and fractionated (e.g., fractionated by pH, molecular size, chemical modifications, and extraction in organic solvents) HAs from different sources (ores, soils, and vermicomposts) to a variety of monocot (e.g., *Zea mays* L. and *Oryza sativa* L.) and dicot (e.g., *Arabidopsis thaliana*) species under various growth conditions (e.g., hydroponics and pot culture) using both soil and foliar applications. However, these studies have failed to elucidate a direct causal relationship between a HS chemical structure(s) and elicitation of the PBR.

The failure to elucidate the chemical structure(s) responsible for the HS-elicited PBR is unquestionably due to (1) the chemical complexity of HS, which consist of a wide variety of chemicals of both plant and microbial origin, (2) the difficulty of isolating fractions of similar chemical identity, and (3) the possibility that a variety of chemical structures might be capable of eliciting the PBR. The proposed conclusion from these studies is that low-molecular-weight hydrophilic molecules that are trapped in hydrophobic domains, when released to the soil solution, can react with the plant plasma membrane (PPM) or enter the cytoplasm and elicit the PBR. Such molecules are hydrophilic due to the presence of one or more or both carboxylic acid or phenolic hydroxyl functional groups. Phenols and polyphenols are naturally produced by plants and a range of activities have been attributed to them, most notably antioxidant activity (Naikoo et al., 2019; Sharma et al., 2019; Chen et al., 2020). Reports on the involvement of COOH groups in the HS PBR are mixed. In one study, COOH acidity and total acidity were reported to negatively affect the activity of H^+^ ATPase and root surface area and root dry mass (Canellas et al., 2008). In another study, COOH groups were positively correlated to root surface area, total root length in the 0.5- to 1.5-mm-diameter class and, to a lesser extent, total root length (Garcia et al., 2016). Aguiar et al. (2013) showed that carboxyl content was positively correlated to root growth and claimed that carboxyl functional groups are required for HS bioactivity. Muscolo et al. (2010) found that root length was decreased by COOH-rich fractions but not the number of roots whereas phenolic-rich fractions inhibited both. Interestingly, the potential role of quinones, which are known to be present in HS, in the HS PBR has been virtually ignored since the work of Wolfgang Flaig and others in the mid-20th century (Flaig, 1961). For example, Flaig and Otto (1951), working with water cress, demonstrated that hydroxyanthraquinones used as proxies of quinones present in HS, particularly alizarin (1,2-dihydroxyantraquinone) and quinalizarin (1,2,5,8-tetra hydroxyanthraquinone) applied at a concentration of 1 × 10^−6^ g mL^−1^, stimulated root growth, by 28.9% and 27.9%, respectively, relative to controls. In addition, various molecular weight fractions obtained by dialysis (i.e., <3,500 Da and 3,500–14,000 Da) of several IHSS standard HAs were found to contain high concentrations of quinones relative to the bulk HA, based on reducing capacity per gram of C (Yang et al., 2016). Based on these results, the effect(s) of electron shuttle activities of HA on the PBR deserve further investigation. Unfortunately, most of the structure–activity relationship studies using modern analytical characterization techniques and chemometric analyses, conducted to elucidate the potential role of HS functional groups, have been focused on COOH and ArOH groups and the potential role of quinones has been ignored.

However, to attempt to provide more information on the participation of COOH and phenolic OH (ArOH) functional groups in the HS PBR, we decided to chemically modify them via alkylation or methylation of the COOH groups and acetylation of the ArOH groups. Alkylating/acetylating these groups would have the effect of removing them from any reactions involved in elicitation of the HS PBR. We subjected a purified humic acid (NHA) from an oxidized sub-bituminous coal to (1) an acetylation reaction to protect just ArOH groups (OHA), (2) an esterification reaction to methylate the COOH groups (AHA), and finally (3) a methylation reaction to protect both ArOH and COOH groups (FHA). The 4 HA along with control (i.e., no HA) were then investigated in a *Z. mays* L. (corn) seedling bioassay to determine what effects alkylation of the ArOH and COOH groups had on the NHA-elicited PBR by assessing seedling biomass and morphological characteristics, when grown in both nutrient-stressed and non-stressed conditions.

## 2 Materials and methods

### 2.1 Isolation and purification of humic acid

Humic acid (HA) was extracted and purified from an oxidized sub-bituminous coal, obtained from northwest New Mexico, according to the new standardized method (Lamar et al., 2014) with modification in the extract ore weight-to-volume ratio. Briefly, approximately 50 g of ore was milled to pass a 60-mesh sieve. The HA (25 g) was extracted in 1 L of 0.1 M NaOH for 6 h under N_2_. After extraction, the entire volume was centrifuged at 4,300 rpm for 60 min to remove insoluble material. The alkaline supernatant was then transferred to a 1-L Erlenmeyer flask and acidified to pH 1 with 37% HCl to precipitate the HA. After *ca.* 2 h to allow full precipitation of the HA, the entire volume was centrifuged at 4,300 rpm for 30 min. After centrifugation, the supernatant (i.e., the fulvic fraction) was decanted, and the precipitated HA was transferred to several 250-mL centrifuge tubes. The HA was then de-ashed in 200 mL of a dilute HCl/HF solution [(5 mL HCl 36% + 5 mL HF 48%) L^−1^] per tube placed on a shaker for 24 h. After two HCl/HF treatments, the HA was washed twice with DI H_2_O acidified to pH 1 with concentrated HCl. After washing, the purified HA was suspended in 50 mL of DI H_2_O, frozen at −80°C, and lyophilized. The lyophilized HA was then stored in a 50-mL centrifuge tube in a desiccator under vacuum until use.

### 2.2 Materials

The following chemicals were purchased from Sigma-Aldrich and used without any further purification: dimethyl sulfate, potassium disulfite, HCl, HF, potassium hydroxide, sodium hydroxide, sulfuric acid, acetic anhydride, methanol, and thionyl chloride. Hoagland’s nutrient solution chemical mix was obtained from Phytotech Labs.

### 2.3 Alkylation/acetylation procedures

The purified HA (NHA) was subjected to alkylation reactions, as detailed below. The reactions were specific to COOH and ArOH groups to produce HA that had selectively blocked carboxylic acids (AHA), phenolic hydroxyls (OHA), and both carboxylic and phenolic hydroxyls (FHA). The HAs were characterized extensively using elemental, ^13^CNMR, FTIR, FT-ICR-MS, and EPR analyses, and TEAC antioxidant potential.

**Phenolic hydroxy acetylation (OHA):** The NHA was ArOH acetylated using a previously reported acetylation procedure (Andjelkovic et al., 2006). Acetylation is a reaction that introduces an acetyl functional group (acetoxy group, CH_3_CO) into an organic chemical compound—namely, the substitution of the acetyl group in place of a hydrogen atom. Briefly, 60 mL of acetic anhydride, 0.7 mL of sulfuric acid, and 2.4 g of purified HA was placed in a 250-mL round-bottom flask equipped with a condenser. The reaction mixture was refluxed at 100°C for 4 h. The reaction was allowed to cool to room temperature, then poured into a beaker containing 300 mL of distilled ice water and stirred to hydrolyze any unreacted acetic anhydride. The mixture was vacuum filtrated and washed with cold distilled water. The solid was oven dried at 90°C until a constant weight was achieved. This procedure was repeated two more times to attempt to acetylate all the hydroxyls of the HA.

**Carboxylic acid methylation (AHA)**: The NHA COOH groups were methylated using a well-known and previously reported esterification reaction (Andjelkovic et al., 2006). In summary, a 500-mL three-necked round-bottom flask was equipped with a condenser. Then, 4 g of purified HA was placed into the flask with 200 mL of methanol. The flask was placed in an ice bath and cooled to 0°C, and thionyl chloride was added dropwise over a period of 2 h. The reaction mixture was allowed to warm to room temperature, and then evaporated to dryness using rotary evaporation. Distilled H_2_O was then added to the flask, the mixture was filtered via vacuum filtration, washed several times with distilled H_2_O, and dried to a constant weight at 90°C resulting in a final mass of 4.5 g. This procedure was repeated several times to ensure that all the carboxylic acid groups were esterified.

**Hydroxy and carboxylic acid methylation (FHA)**: The NHA had the ArOH and COOH groups methylated using a common organic reaction that is modified from the original reported method (Mauthner, 1926). First, 250 mL of degassed distilled H_2_O with 4 g of pure HA was added to a dried three-necked round-bottom flask equipped with a condenser and stir bar under argon. Next, 12 g of potassium hydroxide pellets was added and allowed to stir until fully dissolved and cool. A small amount of potassium disulfite (50 mg) was added to inhibit any potential oxidation side reactions. Then, 20 mL of dimethyl sulfate was added dropwise via a syringe over an hour. After allowing the mixture to stir for 90 min, another 10 mL of dimethyl sulfate was added dropwise over 30 min. The mixture was then allowed to stir overnight. The reaction mixture was concentrated by rotary evaporation to remove any residual dimethyl sulfate; the product was collected via vacuum filtration and washed with distilled H_2_O. The dried product was weighed, and the reaction yielded 5.2 g.

### 2.4 Elemental analyses

The NHA, OHA, AHA AND FHA HAs were subjected to elemental analyses using a Perkin ElmerNexION ICP-MS using 163 method AOAC 3051A/6020 (**Table 1**). The HAs were also analyzed for percent C, H, N, O using a Thermo Fisher Scientific Flash Smart CHBS/O analyzer (**Table 2**). The largest difference was in the percent C in the FHA (**Table 2**) which was offset by the increase in S in the same HA due to the conditions in the methylation reactions (**Table 1**).

### 2.5 Solid-state ^13^C-NMR analysis of HAs

The purpose of the ^13^C-NMR analysis was to assess and compare the bulk molecular structure, composition, and reactivity of the NHA, OHA, AHA, and FHA analyzed by quantitative estimates of carbon functional groups. The NMR pulse programs applied to the ^13^C nucleus included the multiple-composite pulse cross polarization (mcCPMAS) technique introduced by Klaus Schmidt-Rohr (Duan and Schmidt-Rohr, 2017). The advantage of mcCP compared to other cross-polarization techniques is improved quantitation for rapidly relaxing and remotely protonated carbons. Non-quaternary peak suppression (NQS) experiments were performed with a 70-µs delay for C-H dipolar dephasing. All pulse lengths, power levels, and chemical shift values were calibrated using crystalline glycine as an external standard. The ^13^C NMR spectral areas were integrated in chemical shift regions that correspond to the functional groups of carbon. All spectra were acquired while spinning the sample at 12,000 Hz, and spectral areas were corrected for spinning sideband artifacts.

### 2.6 Attenuated total reflectance–Fourier transform infrared spectroscopy of HAs

Infrared spectra of the four HAs were obtained with a Perkin-Elmer Spectrum Two FTIR spectrometer in attenuated total reflectance (ATR) mode. Freeze-dried HA samples were loaded onto the diamond crystal and clamped to a constant loading of 92 N. All spectra were acquired using 50 scans at a resolution of 4 cm^−1^ from 4,000 to 450 cm^−1^ in absorbance mode. Scans were further processed by first smoothing, followed by ATR and baseline correction using Perkin-Elmer Spectrum software.

### 2.7 Electron paramagnetic resonance spectroscopy

Electron paramagnetic resonance (EPR) was used for the determination of semiquinone-type radical content of HAs. The spectra of the four HAs were obtained with a Bruker ELEXSYS-E500 spectrometer equipped with an NMR teslameter (ER 036TM) at room temperature. X-band spectra were collected using a Bruker ER 4105DR double resonator dedicated to quantitative studies and operating in the TE_104_ mode with a nominal center frequency of 9.7 GHz. During measurements, the standard was held in one cavity while the sample was held in the second cavity, so during measurements, both cavities had identical conditions. The standard was an IHSS Leonardite HA of known weight that was permanently embedded in a quartz tube. The semiquinone radical concentration of the Leonardite standard was measured using a Bruker alanine pill standard. Two measurements were made for each HA using different subsamples.

### 2.8 Trolox equivalent antioxidant capacity assay of HAs

The method used to determine the antioxidant activity of the four HAs was based on the capacity of the HAs to inhibit the ABTS radical (ABTS^.+^) by using Trolox as a reference standard, as described by Re et al. (1999) and modified by Mingle and Newsome (2020). The ABTS^.+^ radical was generated by a chemical reaction of 25 mL of ABTS (0.7 mM) with 25 mL of K_2_S_2_O_8_ (2.45 mM) and allowed to stand in darkness at room temperature for 1 h to allow for radical formation and stabilization. The working solution was prepared by taking a volume of the radical solution and diluting it in methanol until its absorbance at 734 nm was 0.70 ± 0.03. The standard calibration curve was obtained using 5 points of Trolox dilutions (0–50 µm). Several concentrations for each HA were assayed to determine the optimal one (20 mM C L^−1^). For the assay, 2 mL of the ABTS^.+^ diluted solution and 100 µL of either the sample or the standard solution was added to the measuring cuvette and thoroughly mixed for 30 s, then the absorbance was measured at 734 nm in a Shimadzu UV-1800 spectrophotometer (Shimadzu, Japan). An inhibition percentage in the range of 20%–80% was achieved. Each sample was analyzed in triplicate. The results were expressed as µmol of Trolox equivalents per mg HA.

### 2.9 Determination of the presence of indole acetic acid or other indoles in the NHA

The presence of indole acetic acid (IAA) and other indoles in the NHA was evaluated by Lifeasible Laboratory Shirley, NY using a UPLC-ESI-MS/MS analysis using internal standards for quantification and actual chemical standards for identification. The indole analysis included the following 27 compounds: indole-3-acetyl glutamic acid, 3-indoleacetonitrile, indole-3-acetyl-L-glutamic acid dimethyl ester, indole-3-L-leucine methyl ester, indole-3-acetyl-L-valine methyl ester, indole-3-acetyl glycine, 2-oxindole-3-acetic acid, indole-3-acetyl-L-aspartic acid, N-(3-indolylacetyl)-L-leucine, N-(3indolylacetyl)-L-valine, indole-3-acetly-L-phenylalanine methyl ester, indole-3-acetyl-L-tryptophan, 3-indole acetamide, tryptamine, indole-3-lactic acid, 3-indoleacrylic acid, N-(3indolylacetyl)-L-alanine, L-trytophan, N-3(indolylacetyl)-L-phenylalanine, indole-3-carboxylic acid, 1-O-indol-3acetylglucose, indole-3-acetic acid (IAA), indole-3-butyric acid (IBA), indole-3-carboxaldehyde, methyl indole-3acetate, 3-indolproprionic acid, and indole. For analysis, 15 mg of the freeze-dried and ground NHA was weighed into a 2-mL plastic microtube. One milliliter of methanol/water/formic acid (15:4:1, V/V/V) and 10 µL of internal standard (100 ng mL^−1^) were added to extract auxins. The mixture was vortexed for 10 min, followed by centrifugation for 5 min at 2,000 r min^−1^ at 4˚C. The supernatant was transferred to clean plastic microtubes and evaporated to dryness. The samples were redissolved in 100 µL of 80% methanol (V/V) and filtered through a 0.22-µm filter (Anpel) prior to LC-ESI-MS/MS.

The sample extracts were analyzed by UPLC-ESI-MS/MS using an ExionLC UPLC and an Applied Biosystems 6500 Triple Quadrupole MS. The UPLC conditions included a Waters Acquity UPLC HSS T3 C18 (100 mm × 2.1 mm, 1.8 µm id) column, a solvent system consisting of 0.04% acetic acid (A) and acetonitrile with 0.4% acetic acid (B), a gradient program starting with 5% B (0–1 min) increased to 95% B (1–8 min), 95% B (8–9 min), and finally ramped back to 5% B (9–12 min), using a flow rate of 0.35 mL min^−1^, a temperature of 40˚C, and an injection volume of 2 µL.

Triple quadrupole scans were acquired on a triple quadropole MS, AB 6500+ QTRAPÒ LC-MS/MS system equipped with an ESI Turbo Ion-Spray interface, operating in both positive and negative ion modes and controlled by Analyst 1.6 software (AB Sciex). The ESI source operation parameters were as follows: ion source, turbo spray: source temperature 550˚C, spray voltage (IS) 5,500 V (Positive), −4,500 V (Negative); curtain gas was set at 35.0 psi, DP and CE for individual MRM transitions were done with further DP and CE optimization.

### 2.10 Plant studies

#### 2.10.1 Growth medium preparation

Loamy sand was screened to <1 mm then autoclaved at 134°C at 2.8 MPa for 37 min. After resting for 24 h at room temperature, each batch was autoclaved a second time using the same settings. The loamy sand was then mixed with perlite and fine vermiculite in a 1:1:1 ratio to create the growth medium.

#### 2.10.2 Seed treatment and corn seedling study conditions

A total of 100 seeds of dent corn (*Z. mays* L.) cv. Nothstine Dent OG were sanitized with 0.5% NaClO for 30 min and then sown in flats of the growth medium. At 5 days after sowing (DAS), germinated seeds were selected to retain 50 germinants with the most uniform root and shoot lengths. These selected seedlings were then transplanted into individual 9-cm pots of the growth medium. The data for HA C content from the elemental analysis was used to calculate the amount of each sample needed to prepare 500 mL at a concentration of 80 mg C L^−1^. The calculated mass of each substance was dissolved in 50 mL of 0.1 M sodium phosphate buffer solution at pH 7. The dissolved treatment samples were then placed in a 250-mL Erlenmeyer flask and pasteurized in an oil bath at 66°C for 30 min. The pasteurized samples were diluted with 450 mL of sterile DI H_2_O under a laminar flow hood to generate the final desired concentration of 80 mg C L^−1^.

At 5 DAS, 50 mL of the HAs were applied to the soil surface in a circle around the corn seedlings for each HA × nutrient regime treatment group at a concentration of 80 mg C L^−1^, with the control plants receiving 50 mL of DI H_2_O instead. In a previous study (data not reported), it was determined that corn seedlings grown at 25% pure Hoagland’s solution gave stressed plants whereas seedlings grown at 75% Hoagland’s solution gave non-stressed plants. For this study, two nutrient regimes were used: one using 25% Hoagland’s solution to produce nutrient-stressed plants and the second at 75% Hoagland’s to produce non-nutrient-stressed plants. All corn plants were provided with 100 mL of 25% or 75% Hoagland’s fertilizer on days 4, 7, 11, 14, 19, and 21 DAS. One pot from each of the 10 treatments was located randomly in each of the six rows, leaving a 9-mm gap between every pot. These pots were then placed on growth platforms forming six blocks (i.e., rows) of 10 pots using a randomized complete block design (RCBD) and the pots in each row were rotated one position from front to back every 3–5 days. The growth platforms, which were located in an environmentally controlled growth room, were each equipped with two LED light panels (Mars Hydro FC6500 680 W) suspended 100 cm above the soil surface. The LED panels were light mapped and calibrated to provide an average of 250 mmol PAR at soil height. The plants were grown on a 12-h/12-h diurnal light cycle at a temperature of 25.5°C and 50% RH. There were six plants per HA per nutrient regime (i.e., 25% Hoagland’s-stressed) or (i.e., 75% Hoagland’s-non-stressed) treatment combination and six plants for each of the non-HA-treated control treatments at 25% and 75% Hoagland’s solution.

#### 2.10.3 Plant harvesting and morphological evaluations

Corn seedlings were harvested at 25 DAS. Immediately prior to harvest, plant height and quantum yield were measured. The quantum yield was measured on the horizontal portion of the V-stage leaf using an Opti-Sciences OS30p chlorophyll fluorometer. The fresh roots of each plant were then washed and separated for imaging using an Epson STD4800 scanner and a Regent Positioning System tray at a resolution of 200 DPI. The root images were analyzed using WinRHIZO Pro 2017a software. The software produced parameter data for root length (cm); root length (cm) for the 0- to 0.5-mm-, 0.5- to 1.0-mm-, and 1.0- to 1.5-mm-diameter classes; projected area (cm^2^); surface area (cm^2^); and average diameter (mm). The roots and leaves of each plant were then placed in separate bags and dried in a drying oven before collection of dry biomass measurements.

#### 2.10.4 Statistical analyses

The data for biomass and morphological parameters and chlorophyl were subjected to an ANOVA (*a* = 0.05), and differences among means were detected with Fisher’s LSD (*a* = 0.05) multiple comparison procedure. The software used for statistical analysis was XLStat (Lumivero). These data were also transformed to percent change from controls using the following formula:


[(Treatment parameter measurement-treatment control)/treatment control]∗100 = % difference from control

The resulting data were then subjected to an ANOVA (*a* = 0.05) and means were separated using Fisher’s LSD. Finally, a principal component analysis (PCA) was performed on the plant morphological parameters (i.e., percent difference from control), the percent of C found in various chemical groups from the ^13^C-NMR analysis, the semiquinone radical content, and the TEAC antioxidant data. In addition, a ratio of pro-oxidants to antioxidants (Pro/Anti) was constructed by normalizing both the semiquinone radical content and the TEAC antioxidant values to the highest value and then dividing the normalized semiquinone radical content by the normalized TEAC values. The resulting Pro/Anti (pro-oxidant to antioxidant) ratios were also included in the PCA.

## 3 Results

### 3.1 Elemental analysis

The HAs in general had very low concentrations of all measured metals and salts because of the HCl/HF de-ashing treatment (**Table 1**). The FHA had elevated (i.e., 8%) [K] and [S] because of the use of KOH and K-sulfate in the reaction to methylate the Ar-OH and COOH groups. As a result, the FHA had a lower [C] compared to the other three HAs (**Table 2**). However, this was compensated for in the plant study as all the plants received 80 mg C L^−1^.

**Table 1:** Concentrations of metals and salts in the HAs.

Element	Humic Acid			
	NHA	AHA	FHA	OHA
	%w/w			
Total nitrogen (N)	1.65	1.51	1.24	1.23
Available phosphate (P2O5)	0.014	0.004	0.008	0.002
Soluble potash (K_2_O)	0.012	0.026	8.12	0.001
Calcium (Ca)	0.014	ND	ND	ND
Sodium (Na)	0.047	0.031	0.047	0.002
Sulfur (S)	0.545	1.38	5.91	2.07
Magnesium (Mg)	0.002	ND	ND	ND
Iron (Fe)	0.136	0.019	0.019	0.008
Boron (B)	0.02	0.005	0.004	0.002
Manganese (Mn)	ND	ND	ND	ND
Copper (Cu)	0.006	0.002	0.002	0.002
Zinc (Zn)	0.002	ND	ND	ND
	mg kg^-1^			
Arsenic (As)	ND	0.37	0.39	ND
Aluminum (Al)	157	56.99	11.36	19.9
Barium (Ba)	3.95	0.74	0.92	ND
Cadmium (Cd)	ND	ND	ND	ND
Chromium (Cr)	7.89	209	6.84	4.24
Cobalt (Co)	2.92	0.27	0.26	0.46
Mercury (Hg)	ND	ND	ND	ND
Lead (Pb)	5.43	2.79	0.32	0.66
Nickel (Ni)	7.73	16.86	6.74	2.21
Molybdenum (Mo)	2.12	18.86	2.64	1.65
Selenium (Se)	3.07	ND	1.84	2.09

**Table 2:** Concentrations of N, C, H, S and O in the humic acids (HAs).

Humic Acid	%N	%C	%H	%O
NHA	1.75	63.23	3.77	25.963
OHA	1.27	62.04	3.84	26.166
FHA	0.47	53.88	3.80	26.069
AHA	1.26	61.45	4.35	23.145

### 3.2 ^13^C-NMR analysis

**Figure 1:**
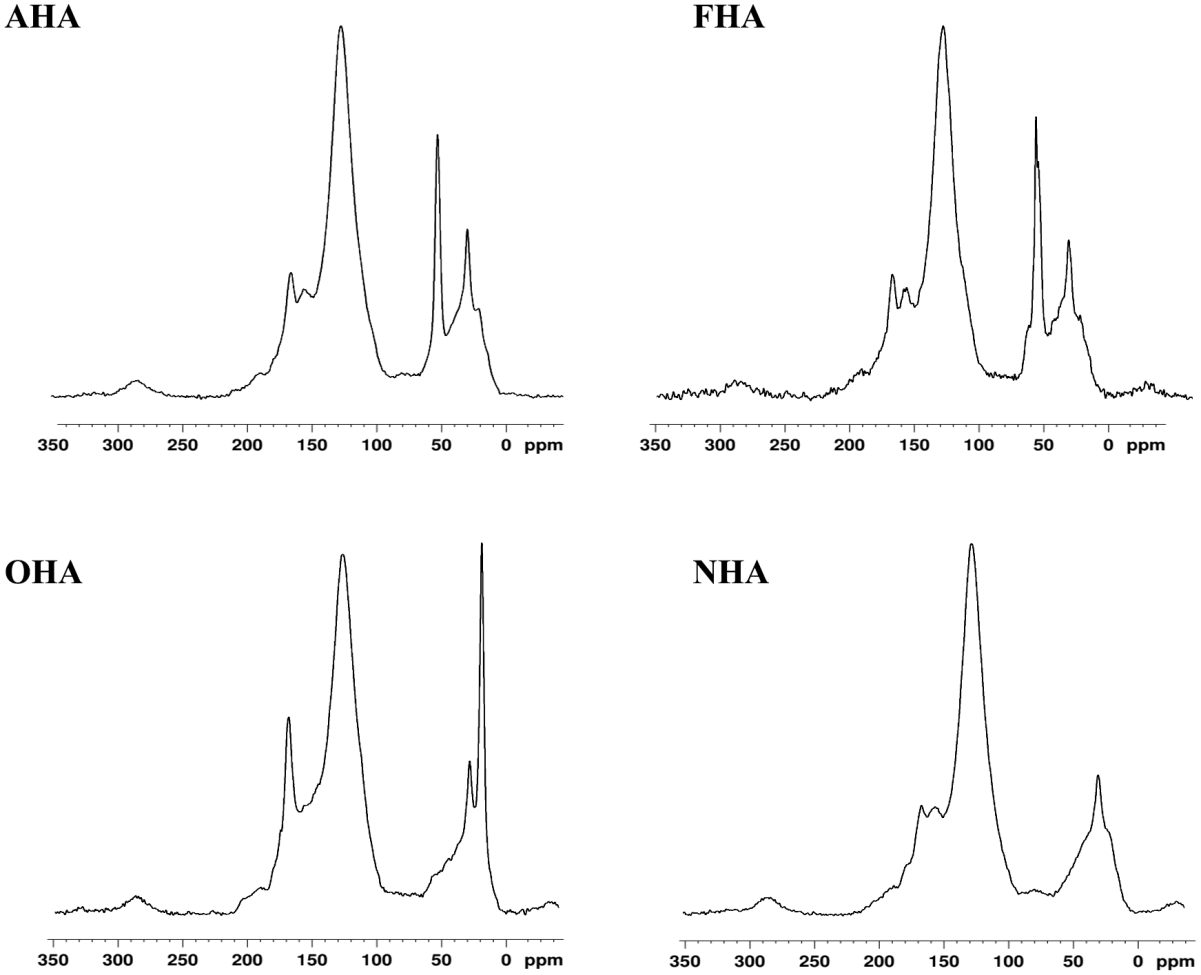
^13^C-NMR Spectra of the original HA (NHA), the ArOH acetylated HA (OHA), the COOH methylated HA (AHA) and the COOH and ArOH methylated HA (FHA).

The ^13^C-NMR analysis supports the significant acetylation/methylation of the phenolic hydroxyl (ArOH) groups and methylation of the carboxylic acids (COOH) groups (**Table 3** and **Figure 1**). In the OHA, alkyl C was increased by 4% (0–50 ppm, **Figure 1**) compared to NHA due to the acetylation of the ArOH groups. In the AHA and FHA, there were 6%–7% increases in the percent of methoxyl C, due to the methylation of both the ArOH and COOH groups. The amounts of aromatic C in the OHA, AHA, and FHA were decreased by 4%–4.5% compared to the NHA due to increases in alkyl C (OHA) and methoxyl C (AHA and FHA) as a result of the acetylation and methylation reactions. Finally, the amount of carbonyl C was increased in the OHA by approximately 4% due to the acetylation of the ArOH groups.

**Table 3:** Percent of C found in chemical shift regions for the HAs.

Chemical Shift		NHA	OHA	AHA	FHA
0–45	Alkyl	15.29	19.30	15.49	15.03
45–60	Methoxyl	3.51	3.34	9.26	10.67
60–95	O-Alkyl	4.32	3.66	4.31	5.02
95–110	Di-O-Alkyl	5.21	4.44	5.07	4.59
110–145	Aromatic	49.00	44.58	45.76	44.49
145–165	Phenolic	12.16	11.86	11.25	10.87
165–215	Carbonyl	8.51	12.81	8.86	9.32

### 3.3 Attenuated total reflectance–Fourier transform infrared analysis

**Figure 2:**
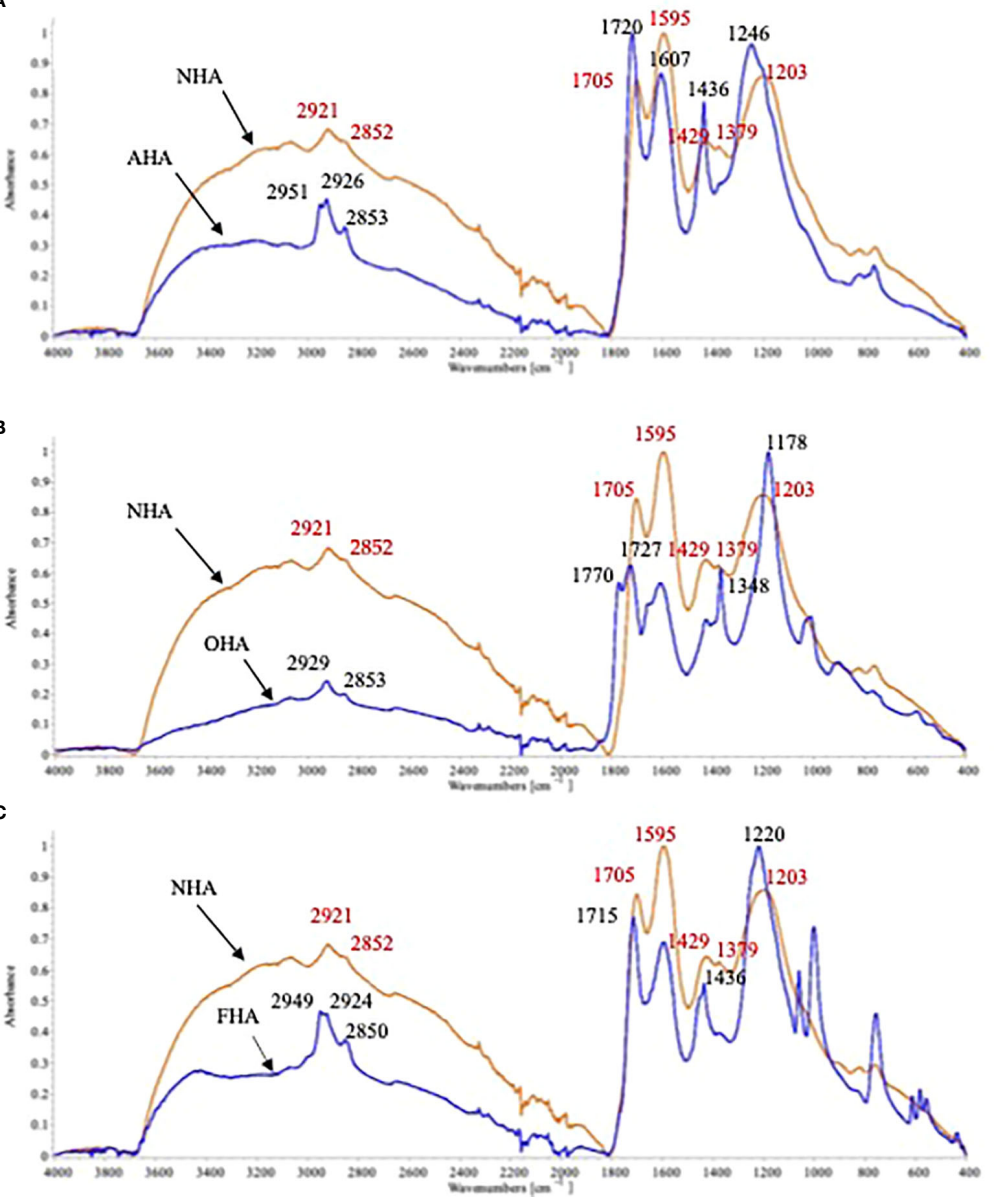
ATR-FTIR spectra of the original HA (NHA) with the **(A)** with acetylated COOH (AHA); **(B)** with methylated ArOH groups (0OHA), and ;**(C)** with methylated ArOH and COOH groups (i.e. FHA).

The NHA spectrum showed the typical HS broad bands at 3,100–3,700 cm^−1^ and 2,400–3,100 cm^−1^, which represent H-bonded OH groups in phenols and carboxylic acids, respectively (**Figures 2A–C**) (Stevenson, 1982). The peaks at 2,921 cm^−1^ and 2,852 cm^−1^ represent the C-H stretch in -CH_3_ and -CH_2_ (Lambert et al., 1987). The sharp peak at 1,705 cm^−1^ is characteristic of C=O of carboxylic acids and the peaks at 1,595 cm^−1^ and 1,379 cm^−1^ represent the antisymmetric and symmetric stretch of COO- in carboxylic acid salts (Lambert et al., 1987). The peak at 1429 cm^−1^ represents the C-H stretch in carboxylic acids (Lambert et al., 1987). Finally, the large peak at 1,203 cm^−1^ represents C-O stretching vibrations in phenols/alcohols (Lambert et al., 1987).

The complexities of spectra of the HA with protected groups, as can be seen from the increased number of peaks in all three spectra of the chemically modified HAs, was increased by the two alkylation and one acetylation reactions. The AHA was produced by esterification of the COOH groups by treatment with thionyl chloride in methanol. This methylation resulted in increases in the peaks at 2,926 cm^−1^ and 2,853 cm^−1^ and the appearance of an additional peak at 2,951 cm^−1^ due to C-H stretching of the added -CH_3_ groups. In addition, the broad peak at 2,400–3,100 cm^−1^ was greatly reduced due to the elimination of H-bonding by the methylated COOH groups. The C=O peak at 1,705 cm^−1^ was replaced by a C=O peak at 1,720 cm^−1^ due to the esterification of the COOH groups (Wagner and Stevenson, 1965; Liotta, 1979). The peaks at 1,607 cm^−1^ and 1,436 cm^−1^ are due to aromatic C=C stretching bands (Earnst and Truong-Son, 2015). Finally, the peak at 1,246 cm^−1^ was due to the C-O stretching of methyl esters (Wagner and Stevenson, 1965).

The acetylation of the OH groups used to produce the OHA had the effect of diminishing the size of both broad bands between 3,100–3,700 cm^−1^ and 2,400–3,100 cm^-1^, indicating that acetylation interrupted H-bonding in general. The peaks at 2,929 cm^−1^ and 2,853 cm^−1^ due to C-H stretching were not greatly affected. There were new peaks produced at 1,770 cm^−1^ and 1,727 cm^−1^ due to the C=O stretching of phenolic acetates and cyclic anhydrides (Wagner and Stevenson, 1965). The sharp peak at 1,348 cm^−1^ is due to CH_3_-C deformation of the added acetyl groups (Wagner and Stevenson, 1965). The large sharp peak at 1,178 cm^−1^ could have been produced from the C-O stretching of phenolic and alcoholic acetates and cyclic anhydrides (Wood et al., 1961).

The FHA was produced by methylation of both OH and COOH groups. As a result, the broad peaks at 3,100–3,700 cm^−1^ and 2,500–3,100 cm^−1^ were both greatly reduced, more so for the latter. In addition, the peaks at 2,949 cm^−1^, 2,924 cm^−^1, and 2,850 cm^−1^ were greatly increased due to the C-H stretch of the added CH_3_ groups. The sharp peak at 1,715 cm^−1^ is due to the C=O of the methyl esters, the peak at 1,436 cm^−1^ is due to the C-H stretch of CH_3_ groups, and the large peak at 1,220 cm^−1^, caused by C-O stretching, was sharpened due to methylation (Wagner and Stevenson, 1965).

### 3.4 Semiquinone radical concentrations, antioxidant analysis–trolox equivalent antioxidant capacity, and pro-oxidant-to-antioxidant ratios of the HAs

**Figure 3:**
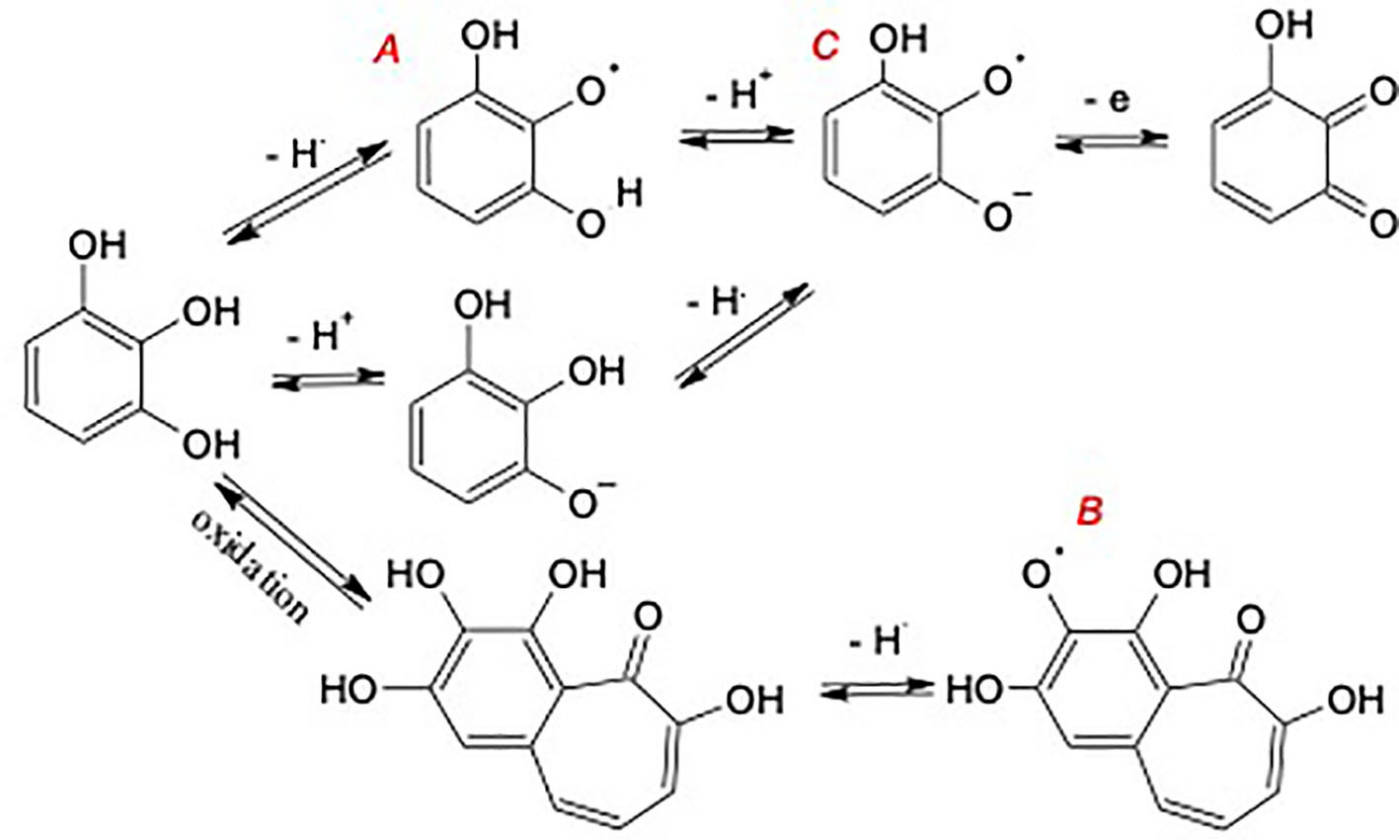
Potential formation pathways of semiquinone radicals (SQR) during the acetylation/alkylation reactions.

Interestingly, the SQR concentration of the NHA was increased by each of the alkylation/acetylation reactions (**Table 4**). The effect was greatest in the AHA where the radical content was increased by 3.4 times, followed by the OHA and FHA, in which the radical concentration was increased by 1.4 times and 1.3 times, respectively. One possible explanation for the increased radical content is the increased hydrophobicity of the HAs due to the acetylation and methylation reactions that allowed O_2_, which is also hydrophobic to diffuse more rapidly throughout the reaction to partially oxidize phenolic moieties (personal communication from Dr. Yiannis Deligiannakis, August 2023). Increases in SQR content could also have been caused by increases in redox equilibria towards oxidation, which might result in shift of phenols towards radical forms A and B but not C in **Figure 3**. The TEAC equivalents were increased in the OHA and decreased significantly in the AHA and FHA relative to that in the NHA (**Table 4**). Owing to its large radical concentration and low relative antioxidant capacity, the AHA was the only HA to have a Pro/Anti ratio greater than 1 (i.e., 1.35) (**Table 4**). The FHA, OHA, and NHA had P/A ratios of 0.74, 0.42, and 0.32, respectively.

**Table 4:** Semiquinone radical contents radical g parameters, TEAC equivalents, and pro-oxidant to antioxidant ratios (P/A) of the HAs. SD = standard deviation.

Humic sample	(x 10^-17^ spins gram^-1^)	SD(x 10^-16^ spins gram^-1^),	g - parmeter	TEAC Trolox Eq mg^-1^	SD	P/A ratio
**NHA**	2.66	4.93	2.0032	1.05	0.05	0.32
						
**FHA**	3.52	0.77	2.0031	0.60	0.15	0.74
						
**OHA**	3.79	2.77	2.0029	1.15	0.15	0.42
						
**AHA**	9.11	6.71	2.0032	0.85	0.00	1.35

### 3.5 Presence of IAA or other auxins in the NHA

Of the 27 indole compounds included in the analysis, the only one that was found in the NHA was indole3-carboxaldehyde (ICAld) at a concentration of 12.37 ng g^−1^ (0.085 nM). This indole, which is also known as indole-3-carbaldehyde and indole-3-aldehyde, is a plant secondary metabolite thought to play a role in plant defense, as a phytoalexin, rather than as a growth regulator and is suggested to occur as a degradation product of IAA (Bottcher et al., 2014). Because ICAld does not possesses a COOH functional group (REF) and it has been identified as a degradation product of IAA (Bottcher et al., 2014) rather than a synthesis intermediate, it is doubtful that it has auxin activity.

### 3.6 Effects of HA treatments on plant biomass and morphological parameters and photosynthesis

Based on seedling heights, root and shoot dry weights, and TRL, the control corn seedlings grown under the nutrient stress regime were significantly shorter, weighed significantly less, and had shorter root systems compared to control plants grown under the non-stressed nutrient regime, indicating that the nutrient stress regime did result in stressful growth conditions. Although the differences were not always significant, all four HA treatments produced taller and heavier seedlings with longer root systems, with few exceptions compared to their respective controls, under both nutrient regimes (**Table 5**). In particular, under the nutrient stress regime, the AHA-treated plants were 14.4% taller and 118.2% heavier (i.e., total dry weight), and had root systems that were 88.5% longer than control seedlings. In comparison, the NHA treatment produced plants that were 6.3% taller and also 118.2% heavier, and had root systems that were 64.7% longer compared to control seedlings. Thus, the AHA treatment produced taller seedlings with more highly ramified roots systems with equivalent dry biomass, compared to NHA-treated seedlings (**Table 5**). The AHA-treated plants also had significantly higher chlorophyll contents than did the NHA- and control-treated plants. Moreover, under nutrient-stressed conditions, the OHA- and FHA-treated seedlings were taller than the NHA-treated seedlings, but all the other measured parameters were relatively equivalent among the three treatments. Thus, removing the ArOH and COOH activities did not appear to affect the HA-stimulated PBR in a negative way. In fact, for AHA-treated plants, the PBR was greater than that produced by NHA-treated plants, although the differences were not statistically different (**Table 5**). A factor that set the AHA apart from the other HAs was the 1.35 P/A ratio, which was less than 1.0 for the other 3 HAs.

**Table 5:** Effect of control and HA treatments on plant biomass, chlorophyll, and morphological parameters.

Treatment	Height (cm)	SDW (g)	RDW (g)	TRL (cm)	TRL 0–0.5mm	TRL 0.5–1 mm	TRL 1–1.5 mm	Chlorophyll
25% Hoagland								
Control	66.6 c	0.92d	0.40 d	2,139.2 e	1,392.2 e	486.9 d	176.9 d	165.2 c
NHA	70.8 bc	2.06 ab	0.82 b	3,522.6 abc	2,281.0 abc	796.4 ab	234.7 ab	183.4 bc
OHA	74.0 ab	1.97 ab	0.83 ab	3,402.9 abc	2,210.4 ab	777.1 ab	254.7 ab	204.9 abc
AHA	76.2 a	2.04 ab	0.84 ab	4,032.6 a	2,643.7 a	910.3 a	251.4 a	238.5 a
FHA	73.6 ab	2.14 a	0.95 a	3,687.9 a	2,372.6 ab	854.9 a	236.5 ab	217.2 ab
75% Hoagland								
Control	71.8 ab	1.32 c	0.59 c	2,656.3 de	1,703.1 de	596.1 cd	221.7 ab	191.6 bc
NHA	75.2 ab	2.06 a	0.82 b	2,910.9 cd	1,839.9 cde	694.4 abc	209.6 bc	186.1 bc
OHA	73.1 abc	1.77 b	0.93 ab	3,543.1 abc	2,206.0 abc	867.6 a	254.7 a	208.6 abc
AHA	70.9 bc	1.77 b	0.83 ab	3,304.3 bcd	2,058.3 bcd	809.9 ab	235.0 ab	190.9 bc
FHA	76.3 a	2.13 a	0.84 ab	3,683.0 ab	2,358.5 ab	852.0 a	255.98 a	192.0 bc

TRL, total root length. Means followed by the same letter are not significantly different at α = 0.05.

In contrast, among plants grown under the nutrient-sufficient regime, the FHA treatment produced the largest plants with the longest root systems (**Table 6**). Furthermore, the OHA-, AHA-, and FHA-treated plants developed longer root systems than did NHA-treated plants (**Table 5**). Finally, the OHA- and the AHA-treated plants had significantly smaller SDWs compared to the NHA- and FHA-treated plants. Of note, the AHA treatment applied under the nutrient stress regime and the FHA treatment applied under the nutrient-sufficient regime, produced the largest plants in the study. However, the longest root systems were produced by AHA-treated plants grown under the nutrient stress regime (**Table 5**). Interestingly, the chlorophyll levels among the HA-treated plants grown under nutrient-sufficient conditions were elevated relative to those found in the respective control plants.

**Table 6:** Effect of HA level treatments on percent difference from controls of biomass, heights, morphological parameters, and chlorophyl content of corn seedlings.

Treatment	Height (cm)	SDW (g)	RDW	TRL (cm)	TRL 0–0.5mm	TRL 0.5–1.0 mm	TRL 1.0–1.5 mm	Chlorophyll
25% Hoagland								
NHA	4.3 bcd	124.9 a	103.3 b	63.3 ab	63.8 d	63.6 ab	32.7 ab	11.0 bcd
AHA	13.3 a	122.6 a	108.0 ab	88.5 a	89.9 bcd	87.0 a	42.1 a	44.4 a
OHA	10.3 ab	115.2 a	105.3 b	59.1 ab	58.8 d	59.6 abc	24.1 bcd	24.0 abc
FHA	9.7 ab	133.2 a	134.0 a	72.4 a	70.4 cd	75.6 a	33.7 bcd	31.5 ab
75% Hoagland								
NHA	4.6 bcd	55.5 b	39.0 c	9.6 c	97.9 abc	16.5 d	−5.4 d	−2.9 d
AHA	−1.3 d	34.3 b	41.2 c	24.4 c	110.9 ab	35.9 cd	6.0 cd	−0.3 cd
OHA	1.7 cd	34.3 b	57.8 c	33.4 bc	119.5 ab	45.6 bc	14.9 abc	8.9 bcd
FHA	6.1 bc	61.2 b	42.4 c	38.7 bc	128.5 a	42.9 bcd	15.4 abc	0.2 cd

TRL, total root length. Means followed by the same letter are not significantly different at α = 0.05.

Treatment percent change from control data for the measured biomass and morphological parameters and chlorophyll are given in **Table 6**. With few exceptions, treatment of plants with any of the HAs resulted in increases in the parameters compared to controls. This was particularly true for RDW and SDW for nutrient-stressed seedlings and fine root growth (i.e., TRL 0–0.5 mm) for the non-nutrient-stressed seedlings (**Table 6**). Percent increases were greater in the seedlings grown under the nutrient stress regime compared to the non-nutrient-stressed seedlings. The fact that the alkylated/acetylated HAs increased corn seedling growth compared to controls to equal or greater extents than the NHA treated seedlings suggests that neither COOH or ArOH groups play significant roles in HA-elicited PBR. Indeed, the magnitude of the PBR of this HA was enhanced by the alkylation/acetylation treatments.

**Figure 4:**
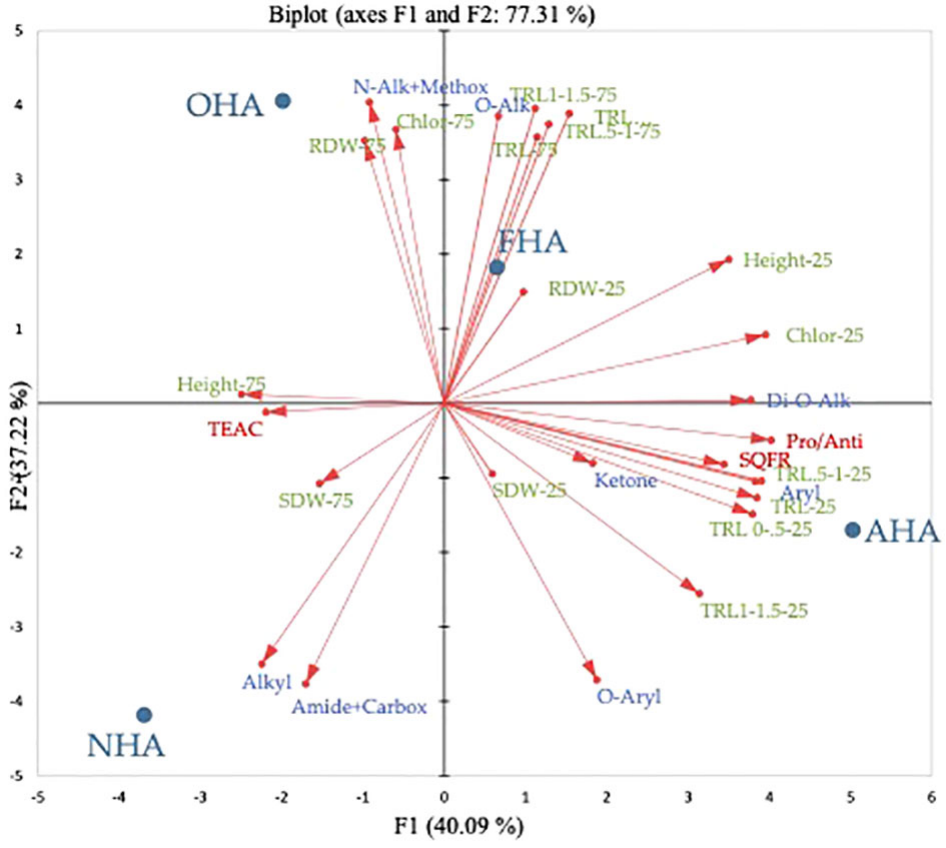
Principal component analysis biplot of the four HAs and the plant parameters, 13C-NMR C types, SQR contents, TEAC equivalents and Pro-oxidant/Antioxidant ratios.

In the full PCA, both principal components (PC) explained about equal amounts of variation for a total of 77.3% of the variation explained by the analysis (**Figure 4**). Across PC1 (i.e., F1) the HA treatments were separated by being COOH-protected HAs (e.g., AHA and FHA) with positive loading values or not COOH-protected (i.e., OHA and NHA) with negative loading values. Principal component 2 (i.e., F2) separated the HA treatments on the basis of being ArOH-protected HAs (e.g., OHA and FHA) with positive loading values, or not (e.g., AHA and NHA), with negative loading values. All of the root length measurements from the seedlings grown under the nutrient stress regime were clustered in the lower right-hand quadrant, which indicates that they were more associated with being affected the most by the AHA treatment, which was COOH-protected but not ArOH-protected. The AHA treatment produced seedlings, grown under the nutrient stress regime, with smaller root systems, by dry weight, but more highly ramified root systems compared to the other treatments (**Table 5**). All the root length parameters had F1 factor loadings of >0.9 with the exception of the TRL 1.0–1.5 parameter with a loading of 0.775. Interestingly, the Pro/Anti ratio, the SQR content and aryl type C also fell in this quadrant and had very strong factor loadings of 0.993, 0.849, and 0.964, respectively. This was consistent with the AHA having, by far, the highest SQR concentration and Pro/Anti ratio and quinones are usually moieties of aromatic compounds e.g., naphthoquinones and anthraquinones, that are known to occur in HS (Berkowitz et al., 1963). The TRL, TRL 0–0.5, TRL 0.5–1, and TRL 1.0–1.5 had F2 factor loadings of 0.891, 0.850, 0.924, and 0.941. Consistent with ArOH protection via methylation (FHA) and acetylation (OHA), methoxyl and O-alkyl type C had F2 loadings of 0.960 and 0.915, respectively. All the root length parameters for the seedlings grown under the non-nutrient stress regime fell into the upper right-hand quadrant and had larger positive factor loadings for F2, which was associated with ArOH-protected HAs, in this case the FHA. The only biomass parameter that had a high loading value was RDW of the seedlings grown under the non-nutrient stress regime, which had an F2 loading of 0.838 and was more closely associated with the ArOH-protected OHA.

## 4 Discussion

The ability of HS to elicit a metabolic reprogramming in plants has been well documented (Roomi et al., 2018) and includes upregulation of important metabolic processes like glycolysis and the tricarboxylic acid cycle (Conselvan et al., 2018), as well as the antioxidant defense (Garcia et al., 2012; Roomi et al., 2018), photosynthetic (Zhang et al., 2013), and nutrient uptake systems (Quaggiotti et al., 2004, also see review by Nardi et al., 2021 and references therein). This reprograming also includes changes to plant morphological characteristics, the most notable and visual of which is the production of more highly ramified root systems, in particular enhanced fine root production (Zandonadi et al., 2007). Enhanced production of fine roots allows HS-treated plants to more thoroughly exploit a given volume of soil for water and nutrients. The complex mixtures of aromatic and aliphatic molecules that comprise HS possess a variety of oxygen-containing functional groups including COOH, ArOH, and quinones that could potentially be involved in eliciting the HS PBR (Stevenson, 1982). The focus of the chemical nature of HS PBR activity has primarily been on the polar and weakly acidic COOH and ArOH groups as well as extents of aromaticity and aliphilicity, possibly because these functionalities and carbon types are the easiest to assess using wet chemistry, for functional groups (Stevenson, 1982), and for C types and functionalities, analytical techniques including ^13^C-NMR (Piccolo et al., 1992) and FTIR (Maluf et al., 2018). Although studied extensively in the mid-1900s, most notably by the German soil organic chemist, Wolfgang Flaig (Flaig and Otto, 1951; Flaig, 1971), the potential roles of quinones and SQR in eliciting the PBR, through redox chemistry, have mostly been ignored. The quinone content of HS can be assessed using wet chemistry techniques (Schnitzer and Riffaldi, 1972), and their potential activities can be measured via EPR to determine the SQR content (Jezierski et al., 2000) or by measuring HS electron-accepting capacity (EAC) and electron-donating capacity (EDC) (Aeschbacher et al., 2010).

In the present study, the focus was on investigating the role of the ArOH and COOH groups in the HS-elicited PBR. An HA, extracted and purified from an oxidized sub-bituminous coal, was chemically modified by alkylation or acetylation, using three different types of reactions to produce three variants of the original HA: the OHA had the ArOH groups protected by acetylation; the AHA had the COOH groups protected by methylation; and the FHA had both the ArOH and COOH groups protected by methylation. The alkylation/acetylation of these groups was designed to eliminate or diminish their participation in the PBR with the hypothesis being that if they were involved in eliciting the PBR, an HA with the activity of these groups reduced or removed would have a reduced or no PBR, as manifested in corn seedling biomass and root system morphological changes. To test this hypothesis, the comparative effects of the three chemically modified HAs (OHA, AHA, and FHA), the NHA and no HA (i.e., control), on corn seedling heights, leaf chlorophyll contents, root and shoot biomass accumulations, and root system morphology, were evaluated under nutrient-sufficient and nutrient stress regimes.

The results of the ^13^C-NMR (**Table 3**, **Figure 1**) and FTIR (**Figure 2**) analysis of the HAs supported extensive alkylation/acetylation of the ArOH and COOH groups, as designed. Even if these groups were not completely alkylated, if they were involved in elicitation of the PBR, partial alkylation/acetylation would have reduced the magnitude of the PBR. However, in general, all three of the differently alkylated/acetylated HA treatments resulted in plants with increased seedling biomass accumulations, heights, chlorophyll contents and root growth equal to or greater than NHA-treated seedlings. These results suggest that either the ArOH and COOH groups may not be involved in elicitation of the PBR or that their activities were increased by acetylation/methylation of the ArOH groups and methylation of the COOH groups.

The ArOH and COOH groups are highly polar and participate in hydrogen bonding and thus contribute greatly to the secondary structure of coal (Liotta, 1979) as well as HS (Cao et al., 2016). Alkylation of these groups has been proposed as a way to try to provide a separation of humic molecules in bituminous and sub-bituminous coals (Liotta, 1979) and lignite HA (Piccolo et al., 2006) in order to achieve better separations that would allow a more complete understanding of HS molecular structures. However, high-pressure size exclusion chromatography of an alkylated Leonardite HA showed a shift in the chromatographic profiles, compared to the original HA, towards smaller elution volumes. This suggested an increase in molecular size that was confirmed by analysis of nominal molecular weight values (Piccolo et al., 2023). However, the apparent increased hydrophobicity of the alkylated molecules may have resulted in molecular aggregation, via hydrophobic interactions, that gave the impression of larger M_w_. In contrast, methylation of Suwannee River fulvic acid (SRFA) prior to analysis by electrospray ionization/mass spectroscopy (ESI/MS) gave smaller Mw than non-methylated SRFA which the authors attributed to disaggregation of larger ions as a result of methylation (Rodstad and Leenheer, 2004). However, prior to ESI/MS, the methylated SRFA was dissolved in a 50/50 solution of methanol/H_2_O to increase the solubility of the alkylated molecules, and that may explain the different results of the two investigations. The alkylation reactions used to produce the OHA, AHA, and FHA HAs would be expected to also render the O-alkylated or acetylated molecules more hydrophobic and therefore less soluble and bioavailable.

The relationship between HA O-alkylated groups and plant growth has been studied previously, both directly, using a methylated vermicompost HA on *Arabidopsis*, corn, and tomato plants by observing effects on root morphology and H^+^-ATPase activity (Dobbss et al., 2010), and indirectly, using PCA to evaluate relationships among C types from ^13^C-CP/MAS-NMR spectra of soil and compost HAs and root morphological parameters from plants treated with those HAs (Garcia et al., 2016). The C content of the methylated vermicompost HA was 8% greater than the original HA, indicating extensive methylation, although no information on whether both ArOH and COOH groups were methylated was provided (Dobbss et al., 2010). However, further use of the same alkylation method demonstrated increases in both alkyl/aryl ethers and esters by FTIR analysis, which indicated methylation of both ArOH and COOH groups (Piccolo et al., 2023). The methylated vermicompost HA promoted significant increases in both lateral root numbers and lengths in *Arabidopsis* and tomato seedlings and lateral root length and H^+^-ATPase activity in corn seedlings compared to the original, vermicompost HA (Dobbss et al., 2010). Lateral root growth stimulation by the methylated vermicompost HA was similar to the results obtained for the FHA and OHA HAs, both of which stimulated lateral root growth in corn seedlings as well as RDW, under non-stress, nutrient-sufficient conditions (**Figure 4**). Based on root growth promotion by methylated vermicompost HA and methylated/acetylated sub-bituminous coal HAs, it appears that neither ArOH nor COOH groups are necessary to elicit a biostimulant response and that alkylated HA can provide a superior response.

The indirect analysis by Garcia et al. (2016) did not use methylated HAs but investigated the effects of a total of 37 HSs and HAs, which were extracted and purified from a variety of Histosols and a vermicompost, on rice root morphology (Garcia et al., 2016). The COOH- and O-alkyl type Cs of the HSs, which contain both HA and FAs, were associated with root surface area and fine root (i.e., 0.5–1.5 mm diameter) length and numbers while aromatic and carbonyl type Cs of HAs were associated with the growth of larger (1.5–3.5 mm and >3.5 mm) roots. The association between COOH type C of the Histosol and vermicompost HS and stimulation of fine root growth was most likely a result of the presence of physiologically active concentrations of the plant hormone IAA in these soils (Szajdak and Maryganova, 2007) and composts (Muscolo et al., 1998, 2009; Schiavon et al., 2010; Trevisan et al., 2010). IAA is known to stimulate root growth at similar concentrations (Zandonadi et al., 2007). In addition, the lack of this association in the purified HAs was probably due to the loss of IAA (M_w_ 175.184 g mol^−1^) and other low-molecular-weight compounds during dialysis of the HA after the HCl/HF wash in Swift’s method, which was employed in this work (Swift, 1996).

**Figure 5:**
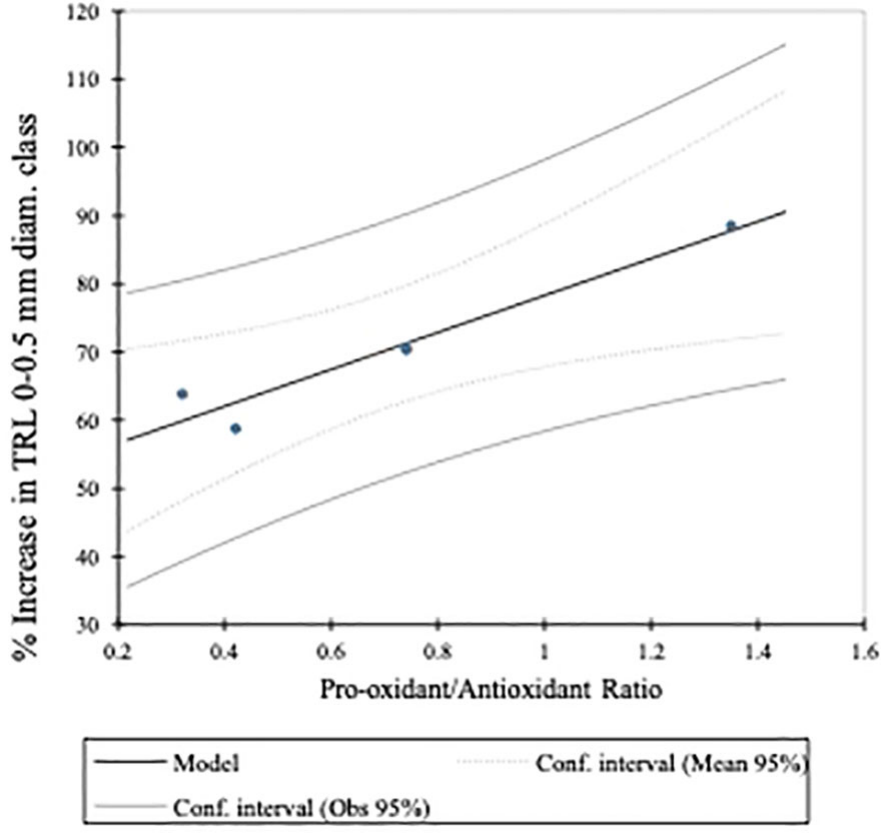
Linear regression of the Pro-oxidant/Antioxidant ratio on the percent increase in TRL of the 0-0.5 mm diameter root class. The Pro/Anti ratio was determined by normalizing both the SQR contents and TEAC equivalents to the highest value among the 4 HAs and dividing the normalized SQRF content by the normalized TEAC equivalent, for each HA.

In corn seedlings grown under the nutrient stress regime, the AHA was associated, very strongly, with TRL of fine (i.e., 0–0.5 mm diameter) roots and TRL (**Figure 4**), the latter of which is mostly composed of the 0- to 0.5-mm-diameter root class (**Table 6**). While the difference was not statistically significant (*a* = 0.05), the AHA treatment stimulated TRL by 15.5% beyond that of the NHA treatment (**Table 5**). The SQR content and the Pro/Anti ratio were also positively and strongly associated with fine root growth and the AHA (**Figure 4**) principally because the AHA had by far the greatest number of SQR and the largest Pro/Anti ratio (**Table 4**). The strong correlation of SQR content and lateral root growth promotion is consistent with the findings of Canellas et al. (2008), who also reported a strong positive Pearson correlation (i.e., 0.82, *p* ≤ 0.05) between SQR and root area, the latter of which is also a measure of lateral root growth (Grover et al., 2021). Moreover, Flaig and Otto (1951), working with water cress, demonstrated that hydroxyanthraquinones, in particular alizarin (1,2-dihydroxyantraquinone) and quinalizarin (1,2,5,8-tetra hydroxyanthraquinone) applied at a concentration of 1 × 10^−6^ g mL^−1^, stimulated root growth, by 28.9% and 27.9%, respectively, relative to controls. As will be discussed later, like SQR, quinones can stimulate production of the superoxide radical. The AHA was the only HA to have a Pro/Anti ratio that was greater than 1 (i.e., 1.5) (**Table 4**). This meant that the AHA possessed more pro-oxidant capacity relative to antioxidant capacity. The Pro/Anti ratio of HAs, that is, the interplay between pro-oxidant activity, which can stimulate ROS production, and antioxidant activity, which can quench ROS, could be a key factor in their biostimulant potential (BP). A plot of the Pro/Anti ratio against the percent increase in TRL 0–0.5 mm roots of the seedlings grown under nutrient stress revealed a strong positive relationship (*R* ^2 =^ 0.939) between the two parameters (**Figure 5**). The strength of this relationship suggests that the ratio of the pro-oxidant activity and the antioxidant activity of HA did indeed play a key role in the concomitant magnitude of the stress response that was elicited and in determining the extent of fine root growth stimulation that resulted from the different treatments, in this case, from eustress. Thus, the greater fine root stimulation observed in AHA-treated corn seedlings might have arisen from additional stress, beyond that produced by the NHA, created by the preponderance of pro-oxidant activity in the AHA as a result of the comparatively high SQR content relative to its antioxidant activity.

Pro-oxidant or EAC originates primarily in HA quinonoid structures (Scott et al., 1998; Aeschbacher et al., 2011), and the antioxidant capacity, or the EDC, originates in HA phenolic structures (Aeschbacher et al., 2012). As pro-oxidants, quinones, semiquinone radicals, and other extracellular electron acceptors (EEA) can be reduced to radical species or, in the case of semiquinone radicals, to hydroquinones, by PPM NAD(P)H oxidoreductase flavoenzymes, which shuttle electrons from cytoplasmic NAD(P)H or ascorbate to the apoplast in order to maintain cytoplasmic redox homeostasis (Van Gestelen et al., 1998; Schopfer et al., 2008; Luthje et al., 2013). The resulting radicals (e.g., semiquinone radicals in the case of quinones) can then reduce molecular oxygen to the superoxide radical (Van Gestelen et al., 1998). This can lead to further reactive oxygen species (ROS) formation [i.e., H_2_O_2_ and hydroxyl radical (HO.)], which can extend and intensify the oxidative stress response through attacks on membrane lipids, proteins (e.g., enzymes), and nucleic acids. For example, in the right side out soybean vesicles, superoxide radical production was found to be enhanced in the presence of several naphthoquinones (NQ), including menadione (Eh = −203 mV), juglone (Eh = −93 mV), 2,3dichloro-1,4 NQ (Eh = −36 mV), and 1,4-NQ (Eh = −140 mV), by PM-bound NAD(P)H-dependent oxidases (Van Gestelen et al., 1998). A second result of the initiation of the transfer of electrons from cytoplasm to apoplast, by quinones, SQR, and other extracellular electron acceptors, for example, ferricyanide (Marre et al., 1988) and the ascorbate radical (Horemans et al., 1994), is a decrease in the electrical contribution of the electrochemical potential gradient across the membrane, resulting in PM depolarization. Interestingly, in support of this mode of action, exogenously applied ferricyanide stimulated elongation and proton extrusion (i.e., H^+^-ATPase activity) in maize and soybean coleoptiles (Carrascoluna et al., 1995) and PM depolarization and medium acidification (i.e., H^+^-ATPase activity) in *Eloda densa* leaves (Marre et al., 1988) in a similar manner to the responses that have been observed in plants exposed to HAs (Zandonadi et al., 2007).

Plant roots possess PM Ca^2+^ channels that are activated by depolarization and referred to as depolarization-activated calcium channels (DACCs) (Thion et al., 1998). As has been shown for HA-elicited PM depolarization (Steinberg et al., 2004), the extent of depolarization is elicitor concentration dependent, with higher concentrations causing greater depolarizations. Upon PM depolarization, the DACCs are activated and allow Ca^2+^ to flow into the cytosol from the apoplast or from intracellular organelles, including vacuoles and endoplasmic reticulum (Feno et al., 2019), causing a transient elevation of the free [Ca^2+^]_cyt_ (White, 2004). The spatiotemporal pattern of the Ca^2+^ flux creates a unique “Ca^2+^ signature” that is proportional to the extent and duration of the PM depolarization, which, in turn, is sensitive to and reflects the concentration of the elicitor (Qudeimat and Frank, 2009). Changes in [Ca^2+^]_cyt_ occur during the transduction of a broad range of biotic and abiotic stresses (Sanders et al., 2002). Increases in apoplast to cytoplasm Ca^2+^ fluxes, in response to exposure of plant roots to HA, have also been reported (Ramos et al., 2015). Additional evidence of HA-elicited Ca^+2^ flux into the cytoplasm has been obtained from *A. thaliana* root cells expressing the red-shifted intensity-based Ca^+2^ RGECO1 reporter, which fluoresces upon exposure to the same NHA used in the current investigation (personnel communication from Dr. Guido Grossman). It is the Ca^2+^ signature, in part, that couples extracellular elicitors, via Ca^2+^ binding/[Ca^2+^]_cyt_ sensor proteins, with specific intracellular responses (Lee and Seo, 2021). These responses most likely include the extensive metabolic reprogramming that has been reported to occur in plants treated with HS (Trevisan et al., 2011) and that are responsible for the eustress response.

## 5 Conclusion

A study was conducted to assess the involvement of ArOH and COOH functional groups in the HA-elicited PBR. A purified sub-bituminous coal HA (NHA) was modified using three different alkylation/acetylation reactions to produce an ArOH acetylated HA (OHA), a COOH methylated HA (AHA), and an ArOH and COOH methylated HA (FHA) to remove or diminish the respective functionalities from participating in elicitation of the biostimulation reaction(s). The comparative PBRs elicited by the 4 HA and control treatments in corn seedlings grown under nutrient stress and nutrient-sufficient regimes were evaluated by assessing their effects on corn seedling heights, biomass accumulations, TRL (TRLs in the 0- to 0.5-mm-, 0.5- to 1-mm-, and 1.0- to 1.5-mm-diameter classes), and chlorophyll contents. With few exceptions, under both nutrient regimes, applications of all 4 HAs resulted in statistically significant increases in corn seedling biomass accumulations and TRLs, compared to controls, and the percent increases were greater in seedlings grown under nutrient stress than they were under nutrient sufficiency. Results for seedling heights and chlorophyll contents were more variable. The fact that the alkylated/acetylated HAs resulted in the same or greater increases in the measured parameters, compared to those produced by NHA-treated seedlings, suggests that neither the ArOH or COOH functionalities may play prominent roles in the HA-elicited PBR, at least for this sub-bituminous coal HA. Furthermore, the alkylation/acetylation reactions produced changes in the HA pro-oxidant capacities by causing increases in the SQR contents and also caused decreases in the antioxidant capacities of the AHA and FHA HAs but an increase in the OHA HA. Linear regression of the ratio of the normalized SQR content, which represents potential ROS-producing capacity, to the normalized TEAC reducing equivalents, which represents potential ROS quenching capacity, and expressed as the Pro/Anti ratio of the four HA on the percent increase in fine root growth (i.e., 0- to 0.5-mm-diameter class) of the corn seedlings grown under nutrient stress conditions, revealed a strong positive (*R* ^2 =^ 0.939) relationship. Because enhanced fine root growth is a reliable indicator of the HA-elicited PBR, the strong relationship between fine root growth stimulation and the pro-oxidant to antioxidant capacities of this HA suggests that this ratio plays a key role in HA BP.

## Data availability statement

The original contributions presented in the study are included in the article. Further inquiries can be directed to the corresponding author.

## Author contributions

RL: Conceptualization, Project administration, Supervision, Writing – original draft, Formal analysis, Investigation, Writing – review & editing. JG: Investigation, Writing – original draft, Writing – review & editing. WH: Formal analysis, Writing – original draft. MJ: Formal analysis, Writing – original draft. HM: Formal analysis, Writing – review & editing.
